# Measurement invariance of the Personality Inventory for DSM-5 across sex

**DOI:** 10.3389/fpsyt.2024.1328937

**Published:** 2024-03-08

**Authors:** Geneviève Rivard, Yann Le Corff, Mélanie Lapalme, Karine Forget

**Affiliations:** ^1^ The Group for Research and Intervention on Children’s Social Adjustment (GRISE), Université de Sherbrooke, Sherbrooke, QC, Canada; ^2^ Département d’orientation Professionnelle, Faculté d’éducation, Université de Sherbrooke, Sherbrooke, QC, Canada; ^3^ Département de Psychiatrie, Faculté de Médecine, Université de Sherbrooke, Sherbrooke, QC, Canada; ^4^ Département de Psychoéducation, Faculté d’éducation, Université de Sherbrooke, Sherbrooke, QC, Canada

**Keywords:** Personality Inventory for DSM-5, maladaptive traits, personality disorders, Alternative Model of Personality Disorders, measurement invariance, scoring approaches

## Abstract

**Introduction:**

There has been an international movement towards dimensional models of personality disorders (PDs) in the last decades, which culminated in the publication of the Alternative Model of Personality Disorders (AMPD) in the Emerging Measures and Models section of the DSM-5. This model was accompanied by a APA-sanctioned Personality Inventory for DSM-5 (PID-5) for the assessment of the AMPD pathological personality traits. One major issue with the assessment of personality disorders pertains to sex differences, and measurement invariance across sex in assessment instruments for PDs is necessary in order to ensure non-biased evaluations and to make valid comparisons between men and women. This study aimed to provide more information on measurement invariance across sex for the PID-5, using both the original scoring approach provided by the authors of the instrument and the scoring approach suggested by the APA in the published version of the PID-5.

**Methods:**

This study was conducted with a sample of 2273 participants from the general Québec (Canada) adult population aged 18 to 90 years (M = 46.59; SD = 16.32; 51.8% women).

**Results:**

The original scoring approach model showed good fit to data after freeing paths between certain traits and reached strict invariance. The APA scoring approach also showed good fit to data and reached strict invariance, but needed an adjustment (path freed between Emotional lability and Impulsivity in men) to reach scalar invariance.

**Discussion:**

In line with previous research, the PID-5 is invariant across sex and the five-factor structure adjusts well to data. The APA scoring approach appears to attenuate the cross-loading problem observed with the original scoring approach. In light of these results, we recommend using the APA scoring approach to derive domain scores.

## Introduction

Models of personality disorders (PDs) presented in international classifications, such as the Diagnostic and Statistical Manual of Mental Disorders, Fifth Edition (DSM-5; 1) and the International Classification of Diseases, Tenth Edition (ICD-10; 2), faced numerous criticisms regarding their validity and their clinical utility over the years (3, 4). In response to these criticisms, there has been an international movement towards dimensional models of personality disorders (PDs). This movement led to the introduction of the Alternative Model of Personality Disorders (AMPD) in the Emerging Measures and Models section of the DSM-5 (1), and five years later a similar model was also published in the ICD-11 (5) that became the official PDs model for all WHO member-states, who were expected to migrate to ICD-11 by January 2022 (6). These new models mark a significant shift in the way PDs are conceptualized (7), that is, from a categorical to a dimensional perspective. In the AMPD, personality disorders (PDs) are defined by two main Criteria: 1) the level of disturbance in self and interpersonal functioning (Criterion A); and 2) a set of 25 maladaptive personality traits (Criterion B) that can be grouped into five domains (8) closely resembling the dimensions of the Big Five model of personality (9). These two Criteria are both necessary to PD diagnosis, but the actual incremental value of one over the other is still subject to debate (10, 11).

With the publication of the DSM-5, the APA AMPD Workgroup developed an official measure to directly index Criterion B which was released with the DSM, the Personality Inventory for DSM-5 (PID-5; 8). The PID-5 is a self-report questionnaire assessing the 25 maladaptive traits as well as the 5 higher order domains, using 220 self-descriptive items (an informant-report version was also developed; 12). These five domains are Antagonism (exaggerated sense of self-importance, antipathy towards others as well as unawareness of others’ needs and feelings), Disinhibition (impulsive behavior motivated by the need of an immediate gratification), Psychoticism (culturally eccentric or unusual behaviors or cognitions), Negative Affectivity (frequent and intense experiences of negative emotions), and Detachment (withdrawal from interpersonal interactions and a limited affective experience and expression). The 25 traits and their associations with the five domains are presented in **Supplementary Table 1** of **Supplementary Materials**.

Since its publication a decade ago, the PID-5 has been translated in many languages, including French (13), and several studies provided strong support for its psychometric properties (14, 15). Most notably, systematic reviews have shown that the PID-5 has good internal consistency, with Cronbach’s alpha coefficients being greater than.80 in most studies for domain scales and greater than .70 for trait scales (14, 15). Studies on its test-retest reliability have shown that domain and trait scores are stable across various time intervals (16–22). The PID-5 was also shown to have good predictive validity, mostly regarding its capacity to predict categorical PDs (15), but also to predict psychosocial functioning (23) and other mental disorders (24, 25). The PID-5 also showed good convergent validity with other traits models at the domain-level, and with other relevant constructs at the trait-level (14, 15).

Several studies investigated the factor structure of the PID-5 (see 26, for a review) using either of the two possible structures/scoring approaches. In the first scoring approach, henceforth labelled the original scoring approach, all 25 trait scales are used as indicators of the five dimensions, while in the scoring approach adopted by the APA (2013; henceforth referred to as the APA scoring approach), only the three most relevant trait scales per domain were retained as indicators (i.e. those with the highest loadings). A meta-analysis on the structural validity of the PID-5 conducted by Watters and Bagby (27) has shown that substantial cross-loadings of traits on two or even more domains and inconsistencies across studies are often observed, especially when using the original scoring approach. Meanwhile, the APA scoring approach appeared to reduce cross-loadings of traits. Despite concerns regarding the two different scoring approaches leading to diverging results, results have shown that they both lead to similar domain scores (28). The APA scoring approach would even be preferable since interstitial traits are removed without losing information in the domains, and fewer items are thus required to access fairly similar information (28).

Despite the numerous validity studies on the PID-5, only five studies (29–33) have investigated its measurement invariance across men and women, even though sex differences are a notable concern in the assessment of PDs (34, 35). Thus, to be able to ensure non-biased evaluations and to make valid comparisons between men and women, we must first ensure that the assessment instruments used are invariant across sex, that is, measurement invariance must be demonstrated (36). In the case of personality traits, measurement invariance assesses the equivalence in terms of structure and of meaning of PD domains across groups (in this study, between men and women) by progressively constraining the factor structure to equality across said groups. Thus, measurement invariance informs whether the same factors (domains) are observed across sex, if these factors are defined by the same indicators (trait scales), if an indicator has the same weight in factor definition and if measurement errors are similar between men and women (37).

Four of the five studies investigating measurement invariance across sex for the PID-5 used shorter versions of the instrument. Two of them (29, 31) used brief versions of the instrument; South et al. (29) used a 36-item version of the PID-5 that assesses the five traditional domains, and a sixth domain of compulsivity, while Gomez et al. (31) used the 25-item version developed by Krueger et al. (38). The structure of the 36-item version was invariant across sex, although strict invariance was not examined (29). The structure of the 25-item version was invariant across sex at the configural, metric and strict levels, but only reached partial scalar invariance. The third study (32), used the PID-5 Faceted Brief Form (PID-5-FBF; 39), a 100-item version, in three distinct samples: populational (n = 526; 49.8% women), private psychotherapy practice clients (n = 544; 64.0% women) and outpatients in a PD treatment clinic (n = 288, 61.5% women). In these three samples, strict invariance (using the original scoring approach) was supported, providing evidence for the absence of sex-related bias in the measure. The last study (33) tested measurement invariance of the PID-5-FBF (39) traits across sex and age in a Belgian community sample (n = 1930; 66.1% women). Of the 25 traits, 10 reached scalar invariance, 13 reached partial scalar invariance, while two reached metric invariance only (Emotional Lability and Separation Insecurity).

In the only study investigating measurement invariance across sex for the original 220-item PID-5 version, Suzuki et al. (30) examined whether its five-factor structure was invariant across men and women in a sample of 6,376 undergraduate students (4195 women, 2181 men) with a mean age of 19.48 years. The authors first conducted exploratory and confirmatory factor analyses (EFA and CFA) to test the proposed structure of the PID-5, using the 25 traits as indicators. The CFA model did not adjust well to the data; therefore an EFA model was tested, leading to a 5-factor model that was similar to the one obtained by Krueger et al. (8) in the development of the PID-5. Then, the authors examined the measurement invariance of this model using exploratory structural equation modeling (ESEM). Results supported the measurement invariance of the exploratory five-factor structure across sex at the configural (invariance of factors), metric (invariance of loadings) and scalar (invariance of intercepts) levels, which indicates that latent means can be compared across men and women (40). Thus, the PID-5 can be considered invariant across sex when using the original scoring approach (30), although no information was provided regarding the APA scoring approach that is sanctioned by the APA and used in research and clinical settings (28).

## The present study

Given that measurement invariance across sex is crucial to make valid comparison between men and women (35), this study aimed to further examine measurement invariance across sex of the PID-5. Since only three studies (30, 32, 33) examined its measurement invariance across sex and used the original scoring approach, the present study aimed to replicate and extend these results by examining measurement invariance for both the original scoring approach and the APA scoring approach. Testing measurement invariance for the APA scoring approach seems particularly relevant, since many clinical decisions rely on it. Furthermore, although Suzuki et al. (30) concluded to scalar invariance, they did not test for strict invariance (invariance of residuals), while Leclerc et al. (32) did test and reach strict invariance but used the 100-item version of the PID-5. Thus, this study aimed to examine invariance of residuals across sex for the 220-item version of the PID-5, since it would inform whether the constructs have the same explicative value across sex (37). Finally, this study used a populational sample of adults that may be more representative of the general population than Suzuki et al. (30) student sample and Leclerc et al. (32) convenience community sample.

## Method

### Participants

The initial sample consisted of 2,505 participants. Since participation was voluntary, took place online without human proctoring, and came with incentives, it is possible that some participants responded carelessly (41). To detect, at least in part, invalid response profiles, long strings analyses were conducted. Participants with more than 32 consecutive identical responses to the PID-5 were removed from the sample. The cut-off for the PID-5 was set based on the fact that, considering the presence of reversed items, the longest possible sequence of coherent identical responses is 32. Therefore, 118 participants (4.7% of the sample) were removed from the sample due to probable careless responding. Additionally, a response inconsistency scale (42) was used to detect inconsistent responding. This scale includes 20 pairs of similar items. The total score represents the sum of the discrepancy between responses to these 20 pairs of items and a score of 17 or more indicates random responding (42). Thus, 117 participants (4.7% of the sample) reached the cutoff value. Only three participants reached the cutoffs for both long strings and inconsistent responding, leading to a final sample of 2273 participants.

Participants were aged between 18 and 90 years (M = 46.59; SD = 16.32; M_men_ = 48.35; SD_men_ = 15.82; M_women_ = 44.94; SD_women_ = 16.62). The sample included 1177 (51.8%) participants whose assigned sex at birth was female, and 1096 (48.2%) whose assigned sex at birth was male. Regarding education, 39.6% (41.8% of men, 37.4% of women) of the sample had a university degree, while 33.7% (29.7% of men, 37.6% of women) had a technical of pre-university college (CEGEP) diploma, 25.6% (27.5% of men, 23.8% of women) had a high school diploma or a specialized trade diploma, and 0.8% (for both men and women) did not complete high school. Regarding marital status, 56.7% (48.8% of men, 51.2% of women) of the sample was married or in a romantic relationship, 32.6% (34.3% of men, 31.1% of women) were single, and 10.4% (8.2% men, 12.5% women) were either widowed, divorced, or separated. Median household annual income was in the 60,000$ to 79,999$ CAN bracket for both men and women.

### Measures

#### Personality Inventory for DSM-5

The French version (13) of the Personality Inventory for DSM-5 (PID-5; 8) was used in the present study. It includes 220 self-reported items rated on a 4-point Likert-type scale ranging from 0 to 3 (0 = very false or often false, 1 = sometimes or somewhat false, 2 = sometimes or somewhat true, 3 = very true or often true). Since the French translation was conducted in Europe and the present study took place in Canada, slight vocabulary adjustments were made to five items to optimize their understandability. Cronbach alphas for the 25 trait scales varied from.72 to.96 for the original version (8), and from.68 to.95 for the French translation in a sample of Belgian, French, and Swiss university students (43). In the present study, they ranged from.75 to.95.

### Procedure

Participants were recruited as part of a larger study on the assessment of the AMPD. The questionnaires were administered via Léger 360, which is the largest Survey firm in Canada. An email invitation was sent to a randomized sample of Léger 360’s panel of over 200,000 residents from the Province of Québec, Canada. Participants under 18 years of age or who reported a “very poor” understanding of the French language were excluded from the study (age and knowledge of the French language were filter questions). The questionnaires were administered online on Léger 360’s secure servers. Participants had the opportunity to save their responses and return to complete the questionnaire at another time. An email reminder was sent to the participants who had not completed the questionnaires one week after the initial invitation. The questionnaire was closed when the target sample size of approximately 2500 completed questionnaires was reached. The data collection began on June 18 and ended on July 15, 2019. Participants who completed the questionnaires received an incentive in the form of points added to their Léger 360 account and that can be exchanged for cash, gifts, or participation in prize draws. There were no missing data in the PID-5 items since the online questionnaire did not allow for missing responses (though missing responses were allowed for sociodemographic questions). The study received approval from the ethics board from the authors’ research institution, and all participants signed an online informed consent form.

### Data analyses

All analyses were conducted using MPlus 8.8 (44). The MLR estimator was used in all factor analyses since it is relatively robust to non-normality, and PID-5 trait scores were not normally distributed in the present sample. To examine the PID-5 factor structure, an exploratory structural equation modeling (ESEM) with target rotation was used. An ESEM was preferred to a confirmatory factor analysis (CFA) because it is less restrictive for a complex model where cross-loadings are expected and theoretically meaningful (45, 46). Target rotation in ESEM allows to target the cross-loadings on specific factors to be as close as possible to zero without applying a constraint; the *a priori* structure can thus be examined without imposing theoretically-unsound constraints on cross-loadings. To assess model fit, values for the Root Mean Square Error of Approximation (RMSEA), Comparative Fit Index (CFI), Tucker-Lewis Index (TLI) and Standardized Root Mean-square Residual (SRMR) were examined. For complex multidimensional models such as the PID-5, Hopwood and Donnellan (47) suggest cutoffs close to.10 for RMSEA, and close to.90 for CFI and TLI to assess model fit. Regarding the SRMR, a value below.08 can be considered acceptable (48). Both the original scoring approach (using the 25 traits as indicators) and the APA scoring approach (using 15 traits as indicators) were tested. Using the two structures obtained with target ESEM, four levels of measurement invariance were then tested: configural, metric, scalar, and strict.

First, the baseline model was tested to examine overall model fit for the whole sample. Then, this baseline model was progressively constrained according to levels of invariance that were reached. At each level, new parameters were constrained to equality to examine if the chosen model is still acceptable in both groups. Configural invariance was tested by fitting an unconstrained multigroup model using the same specifications across sex, thus testing if the model has the same number of factors across men and women. Metric invariance was tested by constraining factor loadings to equality across groups to determine whether each trait contributes to the latent variables (the five domains) similarly in men and women. Scalar invariance was tested by constraining intercepts to equality across groups to determine whether mean differences in the latent constructs capture all mean differences in the shared variance of the traits. Finally, strict invariance was tested by constraining residual variance to equality across groups to determine whether the sum of specific variance and error variance is similar across groups. To determine whether there is measurement invariance, ΔRMSEA and ΔCFI were used, since the Chi-Square test does not perform as well in ESEM as it does in CFA (49). The invariance hypothesis should only be rejected if the decrease in CFI is greater than .01 and the increase in RMSEA is greater than .015 (49). That is, if the supplementary constraints do not significantly decrease the fit of the model, measurement invariance is supported. Moreover, we used the *w*-coefficient to assess the effect size of each additional constraint on the Chi-Square statistic, as it was used in Suzuki et al. (30). The *w*-coefficient can be interpreted according to Cohen’s (50) standards, *w* = .1 representing a small effect size, therefore a small change between compared models (51).

## Results

### Baseline models

Before conducting invariance analyses, baseline models were tested using ESEM with target rotation. The first model to be tested was the five-factor structure with the 25 traits as indicators proposed by Krueger et al. (8). Fit indices showed mixed support for model fit, since the RMSEA, CFI and SRMR suggested an acceptable fit (according to 47 cutoffs) while the TLI indicated inadequate model fit. Modification indices (MI) for all pairs of indicators were examined to identify high measurement error covariances that penalized model fit. Measurement error covariance can indicate the presence of systematic (rather than random) errors and may derive from indicators characteristics such as overlap in content or the presence of a small subfactor within the factor (52). Respecified models were successively computed by freeing the path between Anhedonia and Depressivity (both traits of Detachment; MI = 308.60), Manipulativeness and Deceitfulness (both traits of Antagonism; MI = 327.23), and Rigid Perfectionism (Disinhibition) and Perseveration (Negative Affectivity; MI = 205.91). Following these specifications, the model reached a good fit to the data. Factor loadings for the modified baseline model are presented in **Table 1**.

**Table 1:** Factor loadings – Original scoring approach modified baseline model.

	Negative Affectivity	Detachment	Antagonism	Disinhibition	Psychoticism
Anxiousness	**.783**	.243	-.059	-.042	.019
Emotional lability	**.679**	.055	-.041	.041	.192
Separation insecurity	**.525**	-.030	.176	.187	-.016
Rigid perfectionism	**-.424**	-.246	-.367	.396	-.116
Perseveration	.350	.243	.140	.152	.229
Submissiveness	.346	.101	.160	.201	-.046
Withdrawal	-.074	**.837**	.019	-.034	.108
Restricted affectivity	.342	**-.617**	-.287	-.101	.005
Anhedonia	.235	**.602**	.000	.170	-.004
Intimacy avoidance	-.082	**.522**	.005	.034	.227
Depressivity	.366	.387	-.053	.211	.190
Suspiciousness	.289	.344	.049	.029	.241
Manipulativeness	.033	-.066	**.761**	-.024	.049
Grandiosity	-.040	.012	**.739**	-.246	.206
Deceitfulness	.057	.061	**.597**	.272	.010
Attention seeking	.271	-.357	**.668**	.133	.015
Callousness	-.156	.260	**.584**	.224	.096
Hostility	.296	.277	**.427**	.041	.041
Risk taking	-.294	-.293	.370	.198	.195
Irresponsibility	.037	.152	.195	**.568**	.125
Distractibility	.306	.148	-.077	**.459**	.139
Impulsivity	.239	-.040	.186	.391	.121
Unusual beliefs and experiences	-.082	-.051	.021	-.090	**.946**
Cognitive and perceptual dysregulation	.101	.057	.014	.217	**.673**
Eccentricity	.062	.179	.080	.157	**.521**

N = 2273. Underlined: Traits proposed in the original structure. Bold: Factor loadings equal or greater than.40.

Model fit for the APA scoring approach baseline model had an excellent fit to the data, which allowed to proceed to the invariance analyses. Factor loadings for this baseline model are presented in **Table 2**.

**Table 2:** Factor loadings – APA’s scoring approach baseline model.

	Negative Affectivity	Detachment	Antagonism	Disinhibition	Psychoticism
Anxiousness	**.952**	.112	.010	-.139	-.039
Emotional lability	**.651**	.024	-.019	.043	.165
Separation insecurity	**.506**	-.123	.142	.207	.031
Withdrawal	-.062	**.910**	.012	-.048	-.007
Intimacy avoidance	-.083	**.635**	.046	-.019	.108
Anhedonia	.254	**.582**	-.009	.150	-.065
Manipulativeness	.045	-.048	**.976**	-.084	-.063
Deceitfulness	.043	.053	**.775**	.229	-.092
Grandiosity	-.050	.058	**.541**	-.168	.284
Irresponsibility	-.067	.160	.188	**.612**	.102
Distractibility	.201	.090	-.092	**.583**	.114
Impulsivity	.052	-.037	.092	**.546**	.150
Unusual beliefs and experiences	.010	-.046	.052	-.121	**.926**
Cognitive and perceptual dysregulation	.112	.041	.040	.244	**.616**
Eccentricity	.051	.170	.023	.225	**.497**

N = 2273. Underlined: Traits proposed in the original structure. Bold: Factor loadings equal or greater than.40.

### Measurement invariance across sex

For the original scoring approach model (with the 25 traits as indicators), configural, metric, scalar, and strict measurement invariance were supported. As shown in **Table 3**, changes in RMSEA and CFI were within the recommended thresholds of 0.015 and 0.010, respectively (49). When constraining the number of factors across sex (configural invariance), fit indices were similar to those of the baseline model. Constraining factor loadings across groups (metric invariance) did not significantly reduce model fit. When constraining intercepts across groups (scalar invariance), model fit did not significantly worsen. Finally, when constraining residual variance across sex (strict invariance), fit indices did not significantly change. All *w*-coefficients were lower than 0.1, which indicates that effect sizes for changes in the chi-square statistic were very small when additional constraints were applied.

**Table 3:** Fit indices and invariance test for PID-5 domains.

Invariance test	AIC	BIC	χ^2^	df	χ^2^Men	χ^2^Women	RMSEA	CFI	TLI	SRMR	ΔRMSEA	ΔCFI	*w*
Original scoring approach													
Baseline model	63957.750	64903.011	2980.217	185	–	–	0.082	0.910	0.855	0.027	–	–	–
Modified baseline model^1^	63627.437	64578.427	2674.561	184	–	–	0.077	0.920	0.870	0.026	–	–	–
Modified baseline model^2^	63301.143	64257.862	2321.609	183	–	–	0.072	0.931	0.888	0.025	–	–	–
Modified baseline model^3^	63047.180	64009.628	2096.402	182	–	–	0.068	0.939	0.899	0.025	–	–	–
Configural invariance	62330.734	64255.629	2363.548	364	1245.366	1118.181	0.070	0.936	0.894	0.026	–	–	–
Metric invariance	62391.946	63743.956	2444.821	464	1279.064	1165.757	0.061	0.936	0.918	0.033	-0.009	0.000	0.019
Scalar invariance	62565.541	63802.974	2650.571	484	1400.411	1250.159	0.063	0.931	0.914	0.035	0.002	-0.005	0.066
Strict invariance	62655.733	63749.945	2737.597	509	1464.073	1273.524	0.062	0.929	0.916	0.038	-0.001	-0.002	0.039
APA scoring approach													
Baseline model	42592.631	43136.872	426.898	40	–	–	0.065	0.975	0.935	0.015	–	–	–
Configural invariance	41973.088	43061.570	435.388	80	234.545	200.843	0.063	0.977	0.940	0.015	–	–	–
Metric invariance	42002.369	42804.409	518.623	130	278.822	239.801	0.051	0.975	0.960	0.023	-0.012	-0.002	0.027
Scalar invariance	42213.043	42957.794	796.464	140	439.156	357.307	0.064	0.958	0.937	0.029	0.013	-0.017	0.109
Scalar invariance – modified model^4^	42044.513	42800.722	573.371	138	302.895	270.476	0.053	0.972	0.958	0.028	0.002	-0.003	0.054
Strict invariance	42130.469	42800.745	659.396	153	365.225	294.171	0.054	0.968	0.956	0.033	0.001	-0.004	0.049

N = 2273.

^1^ Paths freed between Depressivity and Anhedonia traits.

^2^ Paths freed between Manipulativeness and Deceitfulness.

^3^ Paths freed between Rigid Perfectionism and Perseveration.

^4^ Paths freed between Emotional lability and Impulsivity in the men group.

Δ : change in fit indices.

- : no data available.

Results for the APA scoring approach (with 15 traits as indicators) are presented in **Table 3**. The configural invariance model had a good fit to the data which was similar to those of the baseline model. Metric invariance was also supported. However, when constraining the intercepts to equality across groups (scalar invariance), the change in CFI was greater than the 0.01 cutoff value suggested by (49). Examination of MI indicated that freeing the path between Emotional lability and Impulsivity in the men’s group could improve the model (MI = 71.92). Following this modification, model fit changes improved, thus allowing to reach (partial) scalar invariance. Finally, strict invariance was supported. Therefore, the PID-5 model based on the APA scoring approach can be considered invariant across sex. All *w*-coefficients were lower than 0.1, which indicates that effect sizes for changes in the chi-square statistic were very small when additional constraints were applied.

Since models are invariant across sex, mean and latent score comparisons between men and women have been calculated for each domain with both the original and the APA scoring approaches. Results are presented in **Tables 4**–**7**. For both scoring approaches, women obtained higher scores on the Negative Affectivity domain and men obtained higher scores for Detachment, Antagonism, and Psychoticism. All effect sizes are small, except for Antagonism for which the difference between men and women is moderate with both scoring approaches. Mean difference in Disinhibition is only significant and of small size with the original scoring approach (men scored higher than women). The same pattern of differences is observed with latent scores.

**Table 4:** Sex comparisons for domain scores – original scoring approach.

Domains	Women (N = 1177)		Men (N = 1096)		t	df	Cohen’s d
	M	SD	M	SD			
Negative Affectivity	1,17	0,42	1,09	0,39	4,39***	2271,00	0,18
Detachment	0,85	0,48	0,90	0,49	-2,50*	2271,00	0,11
Antagonism	0,59	0,42	0,79	0,46	-10,78***	2202,34	0,45
Disinhibition	1,05	0,32	1,11	0,32	-4,36***	2271,00	0,18
Psychoticism	0,62	0,50	0,75	0,53	-5,91***	2232,73	0,25

*p < 0,05 ***p < 0,001.

**Table 5:** Sex comparisons for domain latent scores – original scoring approach.

Domains	Women (N = 1177)		Men (N = 1096)		t	df	Cohen’s d
	M	SD	M	SD			
Negative Affectivity	0,15	0,97	-0,16	0,88	7,93***	2270,03	0,33
Detachment	-0,08	0,92	0,08	0,95	-4,12***	2271,00	0,17
Antagonism	-0,23	0,86	0,25	0,96	-12,69***	2198,05	0,54
Disinhibition	-0,08	0,87	0,09	0,93	-4,57***	2224,45	0,19
Psychoticism	-0,10	0,90	0,11	0,98	-5,35***	2220,40	0,23

***p < 0,001.

**Table 6:** Sex comparisons for domain scores – APA scoring approach.

Domains	Women (N = 1177)		Men (N = 1096)		t	df	Cohen’s d
	M	SD	M	SD			
Negative Affectivity	1,14	0,60	1,00	0,55	5,75***	2270,88	0,24
Detachment	0,87	0,51	0,94	0,52	-3,27**	2271,00	0,14
Antagonism	0,63	0,45	0,84	0,50	-10,62***	2199,98	0,45
Disinhibition	0,79	0,51	0,83	0,51	-1,81	2271,00	0,06
Psychoticism	0,62	0,50	0,75	0,53	-5,91***	2232,73	0,17

***p < 0,001, ** p < 0,01.

**Table 7:** Sex comparisons for domain latent scores – APA scoring approach.

Domains	Women (N = 1177)		Men (N = 1096)		t	df	Cohen’s d
	M	SD	M	SD			
Negative Affectivity	0,11	0,99	-0,12	0,92	5,74***	2270,92	0,24
Detachment	-0,07	0,90	0,08	0,94	-3,91***	2271,00	0,16
Antagonism	-0,19	0,88	0,21	0,98	-10,29***	2199,72	0,43
Disinhibition	-0,03	0,90	0,03	0,92	-1,54	2271,00	0,07
Psychoticism	-0,11	0,90	0,12	0,97	-5,75***	2220,46	0,24

***p < 0,001.

## Discussion

This study examined the measurement invariance of the PID-5 across men and women, using both the original scoring approach, in which the 25 trait scales are used as indicators for the five domains, and the APA scoring approach, in which a reduced sample of the 15 most relevant trait scales are used as indicators. As prerequisite analyses for invariance testing, baseline models were computed and, in line with previous studies, results showed that the five-factor structure of the PID-5 had a good fit to the data with both scoring approaches. In the present study, most of the traits retained for the APA scoring approach had the strongest loadings on their respective domain when using the original scoring approach, which provides support for the APA model. The Antagonism domain appears to be less clearly defined in the original scoring approach, with several traits cross-loading on this dimension, that is, Hostility (λ = .43), Rigid perfectionism (λ = -.37) and Risk taking (λ = .37). This cross-loading of Hostility is consistent with other studies that showed that this trait is theoretically and statistically more associated to Antagonism than it is to Negative Affectivity (53).

Regarding baseline models, while the APA model reached a good fit to the data, adjustments were made for the original scoring approach model to reach an acceptable fit. Paths were successively freed, first between Depressivity and Anhedonia, second between Manipulativeness and Deceitfulness, and lastly between Rigid Perfectionism and Perseveration. In all three cases, these modifications make theoretical sense and may indicate overlap in content between the pairs of traits that is not explained by the factor. Indeed, Depressivity and Anhedonia (Detachment domain) are known to be closely related since the latter is an important aspect of depression (54–56). Similarly, Manipulativeness and Deceitfulness (Antagonism domain) both imply dishonesty and the use of others to one’s benefit. Albeit Manipulativeness is mainly associated with manipulation of others while Deceitfulness is more associated with lying and using others, some items, for example “Sweet talking others helps me get what I want” (item 125, Manipulativeness) and “People don’t realize that I’m flattering them to get something” (item 56, Deceitfulness) are closely related may be responsible for the high MI. Regarding Rigid perfectionism and Perseveration, although they are not part of the same domain, they also are conceptually closely related, since they both imply rigidity and persistence (57). Past studies have shown that Rigid Perfectionism tends to load equally, if not more, onto the Negative Affectivity domain rather than onto the Disinhibition (21, 27, 58).

In accordance with our first hypothesis, strict invariance was supported for the original scoring approach model. This indicates that not only the domain constructs have the same meaning across sex (configural invariance), that traits have an equivalent strength of association to their latent domain construct (factor loadings) across sex (metric invariance), that a same observed score on trait scales indicates a same level on the latent domain variable (scalar invariance), but also that the level of unexplained variations in domain scores are the same across men and women, thus informing that the constructs have the same value and meaning across sex (59). However, the model had to be slightly modified by allowing correlations between pairs of traits, as discussed above.

The second hypothesis was that the five-factor structure of the PID-5 based on the APA scoring approach would be invariant across sex. To our knowledge, invariance for this model had never been tested. While configural and metric invariances were supported, to reach scalar invariance, the path between Emotional Lability (a trait in the Negative Affectivity domain) and Impulsivity (a trait in the Disinhibition domain) were freed, but only in the men’s group. These two traits are both core components of Borderline PD (60), and impulsivity is particularly observed in men (61, 62). Following this respecification of the model, strict invariance was also reached, meaning that constructs have the same explicative value across sex.

Overall, even though both models needed respecifications, the original scoring approach for the PID-5 including 25 traits and the APA scoring approach including 15 traits reached full (strict) invariance across sex. These results are in accordance with other evidence supporting the invariance of personality traits across sex (e.g. 63), and of the level of personality functioning (Criterion A; 34). The APA scoring approach model needed fewer modifications to reach full invariance. This better fit can probably be explained by the removal of interstitial traits, since they seem to be problematic in many structure analyses involving the PID-5 (26). Because the two approaches lead to similar scores, we recommend the use the APA scoring approach, which is the approach used by clinicians, over the original scoring approach when one is interested in using the five domain scores, since it appears to significantly attenuate the cross-loading of traits on domains.

For both scoring approaches, men obtained higher scores on Detachment, Antagonism, and Psychoticism domains, albeit differences were of small effect size, except for the Antagonism domain which had a moderate effect size for both mean and latent score differences. Women obtained higher scores on the Negative Affectivity domain with a small effect size for both mean and latent scores. These findings are consistent with the differences across sex observed by Suzuki et al. (30) for the PID-5, and more broadly with results regarding the Five Factor Model of personality (64). Men obtained significantly higher scores with small effect size on Disinhibition domain, but only with the original scoring approach, for both mean and latent scores. This could be explained by the fact that the Risk Taking trait is not included in the Disinhibition domain score in the APA scoring approach while it is in the original scoring approach; men scored significantly higher than women on Risk Taking with a moderate effect size.

### Limitation and future directions

Although this sample is quite large and is representative of general population, which can provide normative data useful to clinicians and researchers [see (34), for French-Canadian normative data for the PID-5], the proportion of people suffering from severe personality pathology is probably low (as suggested by PID-5 scale scores). This study should thus be replicated in clinical samples. This study also only covers measurement invariance for the self-report version of PID-5. Future studies could assess measurement invariance for the informant version (12), in which both sex of the informant and the assessed could impact on results. Furthermore, this sample is representative of the French-speaking population of the Province of Québec (Canada) only and should be replicated in other cultures, especially non-Western and from developing countries (65). Also, the French version of the PID-5 was used in the present study and results may not generalize to other linguistic versions.

## Data availability statement

The original contributions presented in the study are included in the article/**Supplementary Material**. Further inquiries can be directed to the corresponding author.

## Ethics statement

The studies involving humans were approved by Comité d’éthique de la recherche - Éducation et sciences sociales - Université de Sherbrooke. The studies were conducted in accordance with the local legislation and institutional requirements. Written informed consent for participation in this study was provided by the participants’ legal guardians/next of kin.

## Author contributions

GR: Formal analysis, Writing – original draft, Methodology, Validation, Writing – review & editing. YL: Writing – original draft, Conceptualization, Data curation, Formal analysis, Funding acquisition, Investigation, Methodology, Project administration, Resources, Supervision, Validation, Writing – review & editing. ML: Conceptualization, Data curation, Funding acquisition, Investigation, Methodology, Project administration, Resources, Writing – review & editing. KF: Conceptualization, Writing – review & editing, Data curation, Funding acquisition, Investigation, Methodology.
