# Supplementary material for: Measurement invariance of the Personality Inventory for DSM-5 across sex
Source: Front Psychiatry. 2024 Mar 8;15:1328937. doi: 10.3389/fpsyt.2024.1328937 (PMC10957747; doi:10.3389/fpsyt.2024.1328937)
Supplement: Supplementary file 1 [file Table_1.docx]

| **Table S1**  PID-5 domains and traits | |
| --- | --- |
| Domains and traits | Definition |
| **Negative affectivity** | Frequent and intense experiences of high levels of a wide range of negative emotions and their behavioral and interpersonal manifestations. |
| Emotional lability | Instability of emotional experiences and mood; emotions that are easily triggered, intense, or not proportional to events and circumstances. |
| Anxiousness | Nervousness, tension, or panic in response to various situations; frequent worry about the negative effects of unpleasant past experiences and future negative possibilities; fear of uncertainty; expecting the worst to happen. |
| Separation insecurity | Fear of being alone due to rejection (or separation from) significant others, based on a lack of confidence in one’s ability to care for themselves, both physically and emotionally. |
| Submissiveness | Adaptation of one’s behavior to the interests and desires of others, actual or perceived, even when doing so is contrary to one’s own interests, needs, or desires. |
| Hostility | Persistent or frequent feeling of anger or irritability in response to minor slights and insults; rude, nasty, or vengeful behavior. |
| Perseveration | Persistence on tasks or in a particular way of doing things long after the behavior is no longer functional or effective; continuance of the same behavior despite repeated failures or clear reasons for stopping. |
| Depressivity | See Detachment. |
| Suspiciousness | See Detachment. |
| Restricted affectivity (lack of) | The lack of this facet characterizes low levels of Negative Affectivity. See Detachment for a definition. |
| **Detachment** | Avoidance of socioemotional experience, including both withdrawal from interpersonal interactions and restricted affective experience and expression, especially limited hedonic capacity. |
| Withdrawal | Preference to be alone rather than being with others; reticence in social situations; avoidance of social contacts and activities; lack of initiation of social contacts. |
| Intimacy avoidance | Avoidance of close or romantic relationships, interpersonal attachment, and intimate sexual relationships. |
| Anhedonia | Lack of enjoyment, commitment, or energy for life experiences; deficits in the capacity to feel pleasure and take interest in things. |
| Depressivity | Feeling depressed, miserable, and/or hopeless; difficulty in recovering from such moods; pessimism about the future; pervasive shame or guilt; feelings of inferior self-worth; suicidal thoughts and behaviors. |
| Restricted affectivity | Little reaction to emotionally arousing situations; restricted emotional experience and expression; indifference and aloofness in normatively engaging situations. |
| Suspiciousness | Expectations and sensitivity to signs of interpersonal ill-intent or harm; doubts about loyalty and fidelity of others; feelings of being mistreated, used, or persecuted by others. |
| **Antagonism** | Behaviors that create conflicts with other people, including an exaggerated sense of self-importance and a concurrent expectation of special treatment, as well as a callous antipathy towards others, including both an unawareness of others’ needs and feelings and a willingness to use others in the service of self-enhancement. |
| Manipulativeness | Use of subterfuge to influence or control others; use of seduction, charm, glibness, or ingratiation to achieve one’s ends. |
| Deceitfulness | Dishonesty and fraudulence; misrepresentation of self; embellishment or fabrication when relating events. |
| Grandiosity | Believing that one is superior to others and deserves special treatment; self-centeredness; feeling of entitlement; condescension toward others. |
| Attention seeking | Engaging in behavior designed to attract notice and to make oneself the focus of others’ attention and admiration. |
| Callousness | Lack of concern for others’ feelings or problems; lack of guilt or remorse about the negative or harmful effects of one’s actions on others. |
| Hostility | See Negative Affectivity. |
| **Disinhibition** | Orientation towards immediate gratification, leading to impulsive behavior driven by current thoughts, feelings, and external stimuli, without taking into account past learning or consideration of future consequences. |
| Irresponsibility | Disregard for and failure to honor financial and other obligations or commitments; lack of respect for and lack of follow-through on agreements and promises; carelessness with others’ property. |
| Impulsivity | Acting on the spur of the moment in response to immediate stimuli without a plan or consideration of outcomes; difficulty to establish and follow plans; sense of urgency and self-harming behavior under emotional distress. |
| Distractibility | Difficulty concentrating and focusing on tasks; attention easily diverted by external stimuli; difficulty maintaining goal-focused behavior, including both planning and completing tasks. |
| Risk Taking | Engagement in dangerous, risky, and potentially harmful activities, unnecessarily and without regard to consequences; lack of concern for one’s limitations and denial of the reality of personal danger; reckless pursuit of goals. |
| Rigid Perfectionism (lack of) | Rigid insistence for everything to be flawless, perfect, and without errors or faults, including one’s own and others’ performance; sacrificing of timeliness to ensure correctness in every detail; believing that there is only one right way to do things; preoccupation with details, organization, and order. |
| **Psychoticism** | Exhibition of a wide range of culturally incongruent, odd, eccentric, or unusual behaviors and cognitions. |
| Unusual beliefs and experiences | Belief that one has unusual abilities, such as mind reading or telekinesis, unusual experiences of reality, including hallucination-like experiences. |
| Eccentricity | Odd, unusual, or bizarre behavior, appearance, speech, or thoughts. |
| Cognitive and perceptual dysregulation | Odd or unusual thought processes and experiences, including depersonalization, derealization, and dissociative experiences; mixed sleep-wake state experiences; thought-control experiences. |
| *Note.* Definitions from DSM-5-TR (APA, 2022, p. 899-901). | |
